# Quality of Life 10 Years After Bariatric Surgery

**DOI:** 10.1007/s11695-020-04726-7

**Published:** 2020-06-13

**Authors:** Piotr Major, Tomasz Stefura, Błażej Dziurowicz, Joanna Radwan, Michał Wysocki, Piotr Małczak, Michał Pędziwiatr

**Affiliations:** 1 2nd Department of General Surgery, Jagiellonian University Medical College, Jakubowskiego 2 st., 30-688 Cracow, Poland; 2 Centre for Research, Training and Innovation in Surgery (CERTAIN Surgery), Cracow, Poland; 3 Students’ Scientific Group at 2nd Department of General Surgery, Jagiellonian University Medical College, Cracow, Poland

**Keywords:** Obesity, Bariatric surgery, Sleeve gastrectomy, Gastric bypass, Quality of life

## Abstract

**Purpose:**

Improvement of the quality of life after bariatric surgery is an important outcome of the treatment. Assessing the long-term QoL results provides better insights into the effectiveness of bariatric surgery.

**Materials and Methods:**

This is a cohort study including patients who underwent bariatric surgery between June 2009 and May 2010 in one academic center. Patients underwent either laparoscopic sleeve gastrectomy (LSG) or laparoscopic Roux-en-Y gastric bypass (LRYGB). Overall, 34 patients underwent LSG (52.3%) and 31 patients underwent LRYGB (47.7%). Preoperatively, and after 1 and 10 years, QoL was assessed using two standardized questionnaires: SF-36 and MA-QoLII. After 10 years, 72% of patients filled out these questionnaires.

**Results:**

The global QoL score before surgery was 48.3 ± 20.6. At the 1-year follow-up, the global total QoL score was 79.7 ± 9.8. At the 10-year follow-up, the global total QoL score was 65.1 ± 21.4. There was a significant increase in total QoL between measurements before the operation and 10 years after surgery in the whole study group (*p* = 0.001) and for patients who underwent LSG (*p* = 0.001). There was no significant difference between total QoL prior to surgery and 10 years after for patients who underwent LRYGB (*p* = 0.450).

**Conclusion:**

LSG led to significant improvement in QoL.

## Introduction

The obesity epidemic remains a major health concern, which results in a constantly growing number of patients being treated with bariatric procedures [1, 2]. The effectiveness of bariatric surgery in the treatment of morbid obesity has been established, and for an increasing number of patients, many years have passed since the time of their initial surgery [3, 4]. Re-evaluating the most commonly performed bariatric procedures (laparoscopic sleeve gastrectomy (LSG) and laparoscopic Roux-en-Y gastric bypass (LRYGB)) from a long-term perspective is essential to provide a new insight into their effectiveness [5].

The majority of published studies that present mostly long-term outcomes after bariatric surgery focus only on weight loss and remission of type 2 diabetes mellitus or other obesity-related comorbidities [6–8]. However, a major objective of surgical treatment of obesity, apart from decreasing mortality and morbidity, is to achieve long-lasting improvement in the quality of life (QoL) [8]. QoL has been recognized as an important marker of health for the general population and those with chronic or life-threatening conditions [9]. Patients seek surgical care most often because of impaired QoL, and improvement in QoL is how they usually assess the effectiveness of the treatment [10].

Previous studies have confirmed improvement in QoL in short term after bariatric surgery, but studies rarely include very long-term observations or compare outcomes of LSG and LRYGB [11–15]. Assessing QoL at a more distant time point could help to confirm the durability of the bariatric and metabolic effects after LSG and LRYGB and may reveal potential advantages or disadvantages of these procedures. This study aimed to analyze the effect of bariatric surgery on long-term QoL based on the type of surgery.

## Material and Methods

### Study Design

This is a cohort study that included patients prospectively recruited to the study group, who consecutively underwent bariatric surgery in one academic center between June 2009 and May 2010. Inclusion criteria were providing informed consent to participate in the study and meeting the eligibility criteria for bariatric treatment, either for LSG or LRYGB (body mass index [BMI] ≥ 35 kg/m^2^ with obesity-related comorbidities or BMI ≥ 40 kg/m^2^) [16]. Only patients presenting for primary bariatric procedures were included. Patients undergoing revisional procedures were excluded from the study. The decision concerning the choice of operation (LSG vs. LRYGB) was reached by a patient–surgeon consensus after the patient received extensive medical, dietetic, and psychological consultations. This study includes an intention to treat analysis. There were no revision surgeries or transfers between groups.

### Treatment Protocol

The fast-track pathway was used in the preoperative, intra-operative, and postoperative period. The surgical techniques of LSG and LRYGB were standardized and consistent in the whole study group. A comprehensive description of the perioperative care protocol and surgical technique used in our center can be found in our previous report [17].

### Quality of Life Assessment

The QoL of the study group was assessed at three time points:

1. Pre-surgery QoL assessment: (approximately 3 months before surgery during the qualification process).
2. First follow-up QoL assessment: 1 year after surgery.
3. Second follow-up QoL assessment: 10 years after surgery.

To assess the QoL of the included patients, two licensed and standardized questionnaires were used, which are designed for medical purposes: SF-36 (the Short Form Health Survey) and MA-QoLQII (the Moorehead–Ardelt Quality of Life Questionnaire II).

SF-36 is a self-assessment method. The questions allow assessment of eight indicators of QoL: physical function, role physical, body pain, general health, vitality, social function, role emotional, and mental health. The indicators can be pooled into two scales, physical and mental, or presented as a total score [18].

MA-QoLQII was designed as a part of the Bariatric Analysis and Reporting Outcome System. It includes six parameters to measure QoL: general self-esteem, physical activity, social contacts, satisfaction concerning work, pleasure related to sexuality, and focus on eating behavior [19].

Each postoperative QoL assessment was associated with measurement of weight, BMI, percentage of total weight loss (%TWL), percentage of excess weight loss (%EWL), and percentage of excess BMI loss (%EBMIL).

### Statistical Analysis

All data were analyzed with Statistica version 12.0 PL (StatSoft Inc., Tulsa, Oklahoma, USA). The results are presented as number and percentage, mean with standard deviation (SD), and median with interquartile range (IQR) when appropriate. The Shapiro–Wilk test was used to check for normal distribution of data. To assess the statistical significance of qualitative data differences in subgroups, Pearson’s chi-square or Fisher’s exact when appropriate were used. Quantitative data were analyzed with the Student *T* test, Mann–Whitney *U* test, Kruskal–Wallis ANOVA, and post hoc testing. Results were considered statistically significant when the *p* value was less than 0.05. The influence of baseline characteristics on changes in QoL was analyzed in univariate and multivariate regression models.

## Results

### Material

**Fig. 1:**
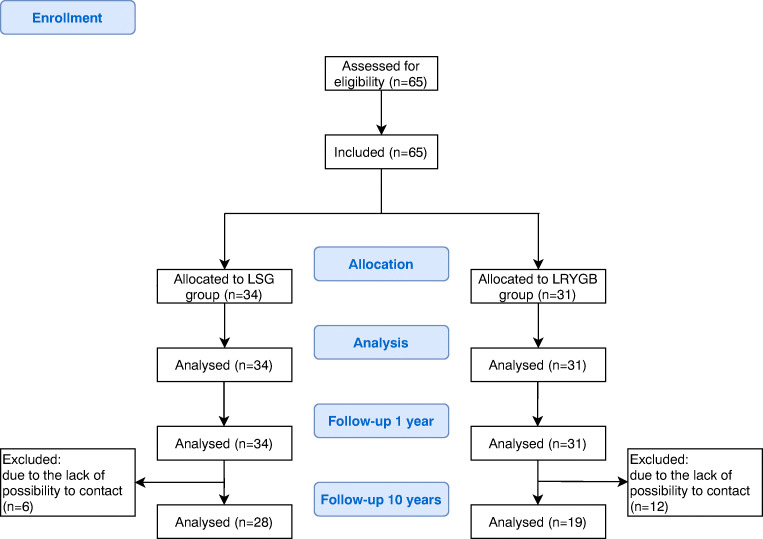
The flow of the study

Initially, the study group included 65 patients undergoing surgical treatment for morbid obesity [39 females (60%) and 26 males (40%)]. Mean age was 42.8 years. Overall, 34 patients underwent LSG (52.3%) and 31 patients underwent LRYGB (47.7%). Mean initial body weight was 146.2 kg, and mean BMI before surgery was 50.4 kg/m^2^. A group of 58 (89.2%) patients was diagnosed with hypertension, 44 (67%) patients were diagnosed with lipid disorders, 34 (52.3%) patients were diagnosed with type 2 diabetes mellitus, 13 (20%) patients were diagnosed with metabolic syndrome, and obstructive sleep apnea occurred in 11 (16.9%) patients (Table 1 and Fig. 1).

**Table 1:** Baseline study group characteristics

Parameter	All	LSG	LRYGB	*p* value
Total, *n* (%)	65 (100)	34 (52.3)	31 (47.7)	–
Sex (females), *n* (%)	39 (60)/26 (40)	22 (64.7)	17 (54.8)	0.456
Median initial body weight, kg (IQR)	146.2 (120.0–157.0)	130.0 (120.0–140.0)	149.5 (124.5–173.5)	0.400
Mean BMI before surgery, kg/m^2^ (range)	50.4 ± 7.3	47.7 ± 5.6	52.6 ± 8.7	0.700
Hypertension, *n* (%)	58 (89.2)	28 (82.4)	30 (96.8)	0.107
Hyperlipidemia, *n* (%)	44 (67.7)	21 (61.8)	23 (74.2)	0.304
T2D, *n* (%)	34 (52.3)	14 (41.2)	20 (64.5)	0.083
Metabolic syndrome, *n* (%)	13 (20)	5 (14.7)	8 (25.8)	0.356
Sleep apnea, *n* (%)	11 (16.9)	6 (17.6)	5 (16.1)	0.999

*LSG* laparoscopic sleeve gastrectomy, *LRYGB* laparoscopic Roux-en-Y gastric bypass, *BMI* body mass index, *T2D* type 2 diabetes

### Weight Loss Effect

Median body weight and mean BMI decreased 1 year after surgery: 86.5 kg (82–105 kg) and 33.2 ± 5.9 kg/m^2^, respectively. One year after surgery, %TWL was 33.3 ± 6.8%, %EWL was 58.7 ± 13.4%, and %EBMIL was 69.7 ± 16.7%. At the 10-year follow-up examination, median body weight was 93.5 kg (82–110 kg) (*p* < 0.001), and mean BMI was 34.8 ± 6.9 kg/m^2^ (*p* < 0.001). Bariatric effect parameters revealed significant weight loss at 10 years since surgery, including %TWL (29.7 ± 11.5%, *p* < 0.001), %EWL (52.1 ± 19.9%, *p* < 0.001), and %EBMIL (61.7 ± 23.2%, *p* < 0.001). Patient bariatric parameters are presented in Table 2.

**Table 2:** Weight-loss effect of LSG vs. LRYGB

Parameter	Type of surgery	Before surgery	1 year after surgery	10 years after surgery	*p* value*
Weight, kg (IQR)	All	146.2 (120.0–157.0)	86.5 (882.0–105.0)	93.5 (82.0–110.0)	0.003
	LSG	130.0 (120.0–140.0)	86.0 (81.0–95.0)	95.5 (90.0–110.0)	
	LRYGB	149.5 (124.5–173.5)	99.0 (82.0–112.5)	87.0 (75.5–111.5)	
BMI, kg/m^2^ ± SD	All	50.4 ± 7.3	33.2 ± 5.9	34.8 ± 6.9	< 0.001
	LSG	47.7 ± 5.6	31.4 ± 4.9	35.7 ± 6.6	
	LRYGB	52.6 ± 8.7	35.8 ± 6.7	33.3 ± 7.4	
%TWL ± SD	All	N/A	33.3 ± 6.8	29.7 ± 11.5	< 0.001
	LSG		34.3 ± 5.5	25.2 ± 9.6	
	LRYGB		31.8 ± 8.4	36.5 ± 11.1	
%EWL ± SD	All	N/A	58.7 ± 13.4	52.1 ± 19.9	< 0.001
	LSG		61.7 ± 11.9	45.4 ± 17.9	
	LRYGB		54.1 ± 14.7	62.2 ± 19.4	
%EBMIL ± SD	All	N/A	69.7 ± 16.7	61.7 ± 23.2	< 0.001
	LSG		74.0 ± 14.6	54.4 ± 20.8	
	LRYGB		63.3 ± 18.2	72.7 ± 23.1	

*LSG* laparoscopic sleeve gastrectomy, *LRYGB* laparoscopic Roux-en-Y gastric bypass, *IGR* inter-quartile ratio, *SD* standard deviation, *BMI* body mass index, *%TWL* percentage of total weight loss, *%EWL* percentage of excess weight loss, *%EBMIL* percentage of excess BMI loss

*P value refers to the LSG vs. LRYGB comparison

### Quality of Life Assessment

**Fig. 2:**
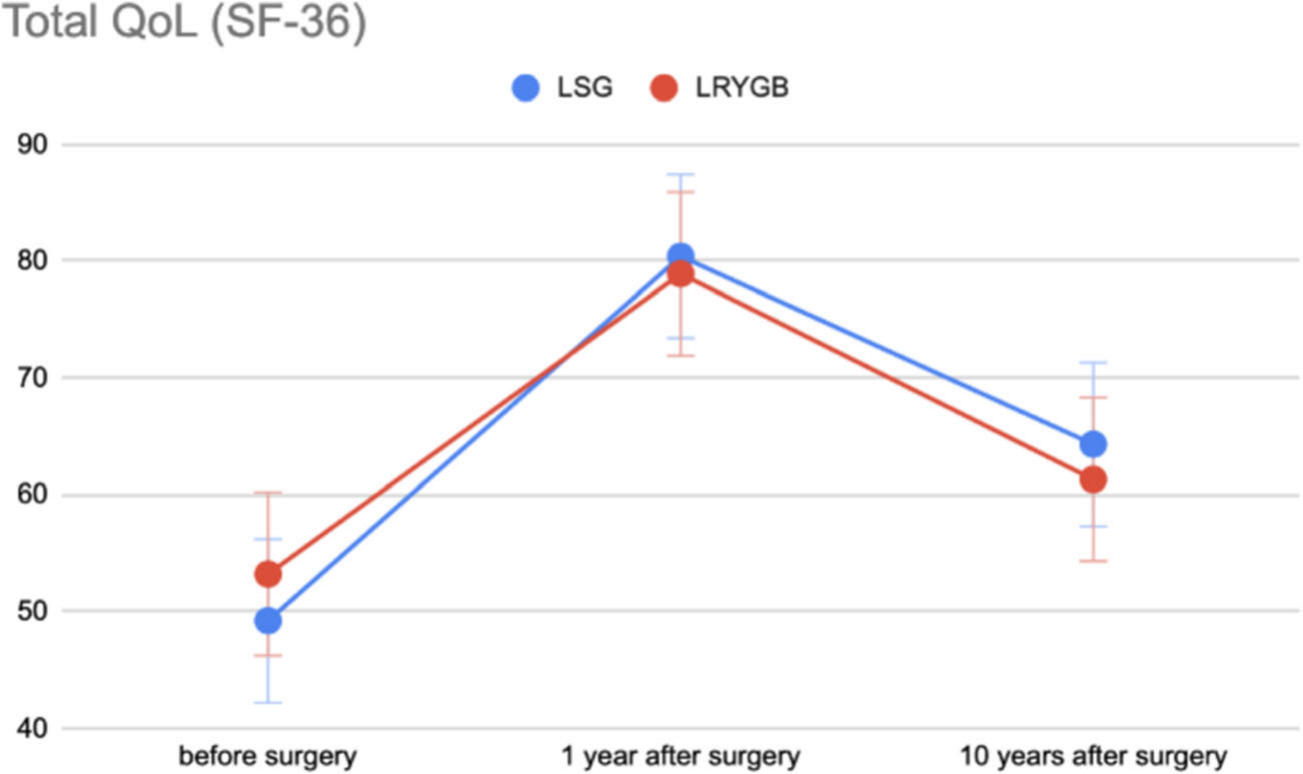
Total QoL after bariatric surgery according to the SF-36 questionnaire

**Fig. 3:**
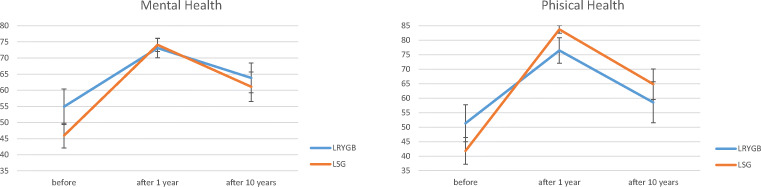
Physical and mental health QoL after bariatric surgery according to the SF-36 questionnaire

According to the results of the SF-36 questionnaire before the surgery, the global QoL related to physical health score was 45.6 ± 20.7 and the global QoL related to mental health score was 49.5 ± 17.7. The global total QoL score before surgery was 48.3 ± 20.6. At the 1-year follow-up, the global QoL related to physical health score was 80.9 ± 11, the global QoL related to mental health score was 73.7 ± 9.3, and the global total QoL score was 79.7 ± 9.8. At the 10-year follow-up examination, the global QoL related to physical health score was 62.3 ± 23, the global QoL related to mental health score was 62.2 ± 17.8, and the global total score was 65.1 ± 21.4. There was no significant difference between LSG and LRYGB in any parameter included in the SF-36 questionnaire (*p* > 0.05). There was, however, a significant increase in total QoL between the measurement prior to the operation and 10 years after surgery in the whole study group (*p* = 0.001) and for patients who underwent LSG (*p* = 0.001). There was no significant difference between total QoL before surgery and 10 years after LRYGB (*p* = 0.450) (Fig. 2). The physical health QoL also increased significantly after the 10-year period for all patients (*p* = 0.003). Subgroup analysis showed that there were significant differences in patients who had undergone LSG (*p* < 0.001), but no changes in patients in the LRYGB group (*p* = 0.678). Measurements of the mental health QoL revealed analogical results, with a significant increase for the whole study group (*p* = 0.006) and the LSG group (*p* = 0.013), with no significant increase in the LRYGB group (*p* = 0.352) (Fig. 3). The results of the QoL measurements using the SF-36 questionnaire are presented in Table 3.

**Table 3:** Quality of life after bariatric surgery (SF-36)

Parameter	Type of surgery	Before surgery (1)	1 year after surgery (2)	10 years after surgery (3)	*p* value (all)	*p* value (LSG)	*p* value +(LRYGB)	*p* value LSG vs. LRYGB*
Global quality of life—physical health, ± SD	All	45.6 ± 20.7	80.9 ± 11	62.3 ± 23	(1–2) < 0.001 (1–3) 0.003 (2–3) < 0.001	(1–2) < 0.001 (1–3) < 0.001 (2–3) 0.005	(1–2) 0.016 (1–3) 0.678 (2–3) 0.108	0.571
	LSG	51.3 ± 22.1	76.5 ± 15.3	58.6 ± 24.5				
	LRYGB	41.8 ± 19.5	83.8 ± 5.8	64.8 ± 22.2				
Global quality of life—mental health, ± SD	All	49.5 ± 17.7	73.7 ± 9.3	62.2 ± 17.8	(1–2) < 0.001 (1–3) 0.006 (2–3) 0.014	(1–2) < 0.001 (1–3) 0.013 (2–3) 0.038	(1–2) 0.02 (1–3) 0.352 (2–3) 0.326	0.159
	LSG	54.9 ± 19.1	73.1 ± 10.4	63.8 ± 16.1				
	LRYGB	45.9 ± 16.2	74.1 ± 8.7	61.1 ± 19.3				
Global—total, ± SD	All	48.3 ± 20.6	79.7 ± 9.8	65.1 ± 21.4	(1–2) < 0.001 (1–3) 0.001 (2–3) 0.007	(1–2) < 0.001 (1–3) 0.001 (2–3) 0.031	(1–2) 0.014 (1–3) 0.450 (2–3) 0.196	0.371
	LSG	54 ± 21	77.3 ± 13.4	63.5 ± 21.7				
	LRYGB	44.6 ± 20	81.4 ± 6.5	66.2 ± 21.7				
Physical function, ± SD	All	45.8 ± 29.4	93.2 ± 11.2	76.3 ± 30.2	(1–2) < 0.001 (1–3) < 0.001 (2–3) 0.03	(1–2) < 0.001 (1–3) < 0.001 (2–3) 0.048	(1–2) 0.011 (1–3) 0.156 (2–3) 0.468	0.207
	LSG	53.8 ± 31.1	88.3 ± 15.7	75 ± 32.4				
	LRYGB	40.6 ± 27.8	96.4 ± 5.1	77.2 ± 29.6				
Role physical, ± SD	All	39.2 ± 4.9	92.5 ± 25.6	68.2 ± 43	(1–2) < 0.001 (1–3) 0.011 (2–3) 0.042	(1–2) < 0.001 (1–3) < 0.001 (2–3) 0.164	(1–2) 0.170 (1–3) 0.992 (2–3) 0.209	0.147
	LSG	47.9 ± 4.5	81.3 ± 38.6	50 ± 48.9				
	LRYGB	33.3 ± 4.2	100 ± 0	80.6 ± 34.9				
Body pain, ± SD	All	54.8 ± 31.1	83.7 ± 19.8	64.7 ± 30.9	(1–2) < 0.001 (1–3) 0.355 (2–3) 0.026	(1–2) 0.002 (1–3) 0.352 (2–3) 0.081	(1–2) 0.135 (1–3) 0.869 (2–3) 0.319	0.825
	LSG	57.1 ± 34.8	81.8 ± 20.5	63.4 ± 34.6				
	LRYGB	53.3 ± 29.1	85 ± 19.7	65.5 ± 29.3				
General health, ± SD	All	40.9 ± 17.7	70.6 ± 13.4	49.1 ± 20.4	(1–2) < 0.001 (1–3) 0.168 (2–3) < 0.001	(1–2) < 0.001 (1–3) 0.11 (2–3) < 0.001	(1–2) 0.064 (1–3) 0.824 (2–3) 0.204	0.180
	LSG	45.7 ± 20.7	65 ± 16.4	50.6 ± 23				
	LRYGB	37.7 ± 15.2	74.3 ± 9.9	48.1 ± 19				
Vitality, ± SD	All	47.8 ± 13.2	64.5 ± 7.6	54 ± 18.7	(1–2) < 0.001 (1–3) 0.206 (2–3) 0.012	(1–2) < 0.001 (1–3) 0.147 (2–3) 0.045	(1–2) 0.124 (1–3) 0.908 (2–3) 0.26	0.424
	LSG	52.5 ± 15	64.6 ± 7.2	55 ± 19.2				
	LRYGB	44.7 ± 11.3	64.4 ± 8	53.3 ± 18.9				
Social function, ± SD	All	57 ± 19.6	74.4 ± 10.4	74.4 ± 24.5	(1–2) 0.002 (1–3) 0.002 (2–3) 0.999	(1–2) 0.026 (1–3) 0.098 (2–3) 0.839	(1–2) 0.071 (1–3) 0.017 (2–3) 0.810	0.344
	LSG	56.5 ± 22.2	75.3 ± 12	80.3 ± 23.9				
	LRYGB	57.3 ± 18.3	73.9 ± 9.4	70.4 ± 24.7				
Role emotional, ± SD	All	50 ± 47	92.2 ± 21	71.1 ± 38.9	(1–2) < 0.001 (1–3) 0.076 (2–3) 0.078	(1–2) < 0.001 (1–3) 0.027 (2–3) 0.273	(1–2) 0.165 (1–3) 0.938 (2–3) 0.293	0.623
	LSG	61.1 ± 48.9	91.7 ± 20.8	66.7 ± 45				
	LRYGB	42.6 ± 45.5	92.6 ± 21.7	74.1 ± 35.4				
Mental health, ± SD	All	51.7 ± 11.9	65.3 ± 9.2	62.5 ± 17	(1–2) < 0.001 (1–3) 0.006 (2–3) 0.688	(1–2) < 0.001 (1–3) 0.022 (2–3) 0.519	(1–2) 0.208 (1–3) 0.184 (2–3) 0.997	0.231
	LSG	58.3 ± 11.1	66.7 ± 8.1	67 ± 15.1				
	LRYGB	47.3 ± 10.6	64.4 ± 10	59.6 ± 18				

*LSG* laparoscopic sleeve gastrectomy, *LRYGB* laparoscopic Roux-en-Y gastric bypass, *SD* standard deviation

*P value refers to a difference from baseline

When analyzing only 19 patients from the LRYGB group who participated in the second follow-up examination, there was no significant improvement in the global total QoL 1 year after surgery (*p* = 0.001). When comparing the baseline score or first follow-up with results obtained at 10 years, no significant difference was observed (Table 4).

**Table 4:** Repeated measures for 19 LRYGB patients who were participating in the second follow-up

Parameter	Before surgery (1)	1 year after surgery (2)	10 years after surgery (3)	*p* value (LRYGB)
Global quality of life—physical health, ± SD	51.33 ± 22.07	76.50 ± 15.32	58.58 ± 24.53	(1 vs. 2) 0.001 (1 vs. 3) 0.467 (2 vs. 3) 0.019
Global quality of life—mental health, ± SD	54.92 ± 19.06	73.08 ± 10.36	63.83 ± 16.07	(1 vs. 2) 0.002 (1 vs. 3) 0.148 (2 vs. 3) 0.130
Global—total, ± SD	54 ± 21.01	77.25 ± 13.36	63.5 ± 21.67	(1 vs. 2) 0.001 (1 vs. 3) 0.222 (2 vs. 3) 0.053
Physical function, ± SD	53.75 ± 31.12	88.33 ± 15.72	75 ± 32.4	(1 vs. 2) 0.001 (1 vs. 3) 0.047 (2 vs. 3) 0.269
Role physical, ± SD	47.92 ± 44.54	81.25 ± 38.62	50 ± 48.85	(1 vs. 2) 0.073 (1 vs. 3) 0.989 (2 vs. 3) 0.097
Body pain, ± SD	57.08 ± 34.83	81.83 ± 29.55	63.42 ± 34.57	(1 vs. 2) 0.079 (1 vs. 3) 0.829 (2 vs. 3) 0.227
General health, ± SD	45.67 ± 20.74	65.00 ± 16.4	50.58 ± 22.99	(1 vs. 2) 0.008 (1 vs. 3) 0.680 (2 vs. 3) 0.054
Vitality, ± SD	52.5 ± 15	64.58 ± 7.22	55 ± 19.19	(1 vs. 2) 0.044 (1 vs. 3) 0.856 (2 vs. 3)0.126
Social function, ± SD	56.5 ± 22.17	75.25 ± 12.02	80.33 ± 23.91	(1 vs. 2) 0.015 (1 vs. 3) 0.002 (2 vs. 3) 0.690
Role emotional, ± SD	61.08 ± 48.91	91.67 ± 20.77	66.67 ± 44.97	(1 vs. 2) 0.094 (1 vs. 3) 0.915 (2 vs. 3) 0.194
Mental health, ± SD	58.33 ± 11.11	66.67 ± 8.06	67.00 ± 15.08	(1 vs. 2) 0.091 (1 vs. 3) 0.077 (2 vs. 3) 0.996

*LRYGB* laparoscopic Roux-en-Y gastric bypass, *SD* standard deviation

Regression analysis of factors potentially influencing changes in the SF-36 score measured 1 year after and before surgery did not reveal significant factors (Table 5). Univariate regression analysis of factors potentially influencing changes in the SF-36 total score measured 10 years after and before surgery revealed hypertension (*p* = 0.041) and hyperlipidemia (*p* = 0.015) to be factors negatively influencing the SF-36 total score. Multivariate analysis did not reveal significant factors (Tables 5 and 6).

**Table 5:** Regression models of factors potentially influencing difference of SF-36 score measured 1 year after and before surgery

	Parameter ± SD	*p* value
Females vs. males	2.03 ± 4.1	0.624
LSG vs. LRYGB	6.79 ± 3.63	0.072
BMI	− 0.03 ± 0.53	0.951
T2D	0.36 ± 3.78	0.096
Insulin therapy	0.87 ± 3.77	0.820
Complications of T2D	− 0.89 ± 6.28	0.889
Hyperlipidemia	− 0.08 ± 4	0.985
Non-alcoholic fatty liver disease	4.09 ± 3.73	0.282
Hypertension	− 5.83 ± 3.96	0.153
Other cardiovascular comorbidity	− 2.17 ± 8.99	0.811
Chronic obstructive pulmonary disease	− 1.45 ± 10.10	0.887
Obstructive sleep apnea	0.3 ± 10.1	0.977

*SD* standard deviation, *LSG* laparoscopic sleeve gastrectomy, *LRYGB* laparoscopic Roux-en-Y gastric bypass, *BMI* body mass index, T2D type 2 diabetes

**Table 6:** Regression models of factors potentially influencing difference of SF-36 score measured 10 years after and before surgery

	Parameter ± SD	*p* value
Univariate		
Females vs. males	5.06 ± 4.71	0.289
LSG vs. LRYGB	6.08 ± 4.35	0.173
BMI	− 0.49 ± 0.61	0.428
T2D	− 6.84 ± 4,22	0.116
Insulin therapy	− 5.2 ± 4.3	0.236
Complications of T2D	0.48 ± 7.35	0.948
Hyperlipidemia	− 9.3 ± 4.33	0.041
Non-alcoholic fatty liver disease	2.06 ± 4.43	0.645
Hypertension	− 11.17 ± 4.32	0.015
Other cardiovascular comorbidity	− 10.73 ± 10.57	0.319
Chronic obstructive pulmonary disease	6.01 ± 11.03	0.591
Obstructive sleep apnea	− 16.99 ± 11.03	0.136
Multivariate		
Hyperlipidemia	− 10.87 ± 9.42	− 0.259
Hypertension	− 17.17 ± 9.69	0.088

*SD* standard deviation, *LSG* laparoscopic sleeve gastrectomy, *LRYGB* laparoscopic Roux-en-Y gastric bypass, *BMI* body mass index, *T2D* type 2 diabetes

**Fig. 4:**
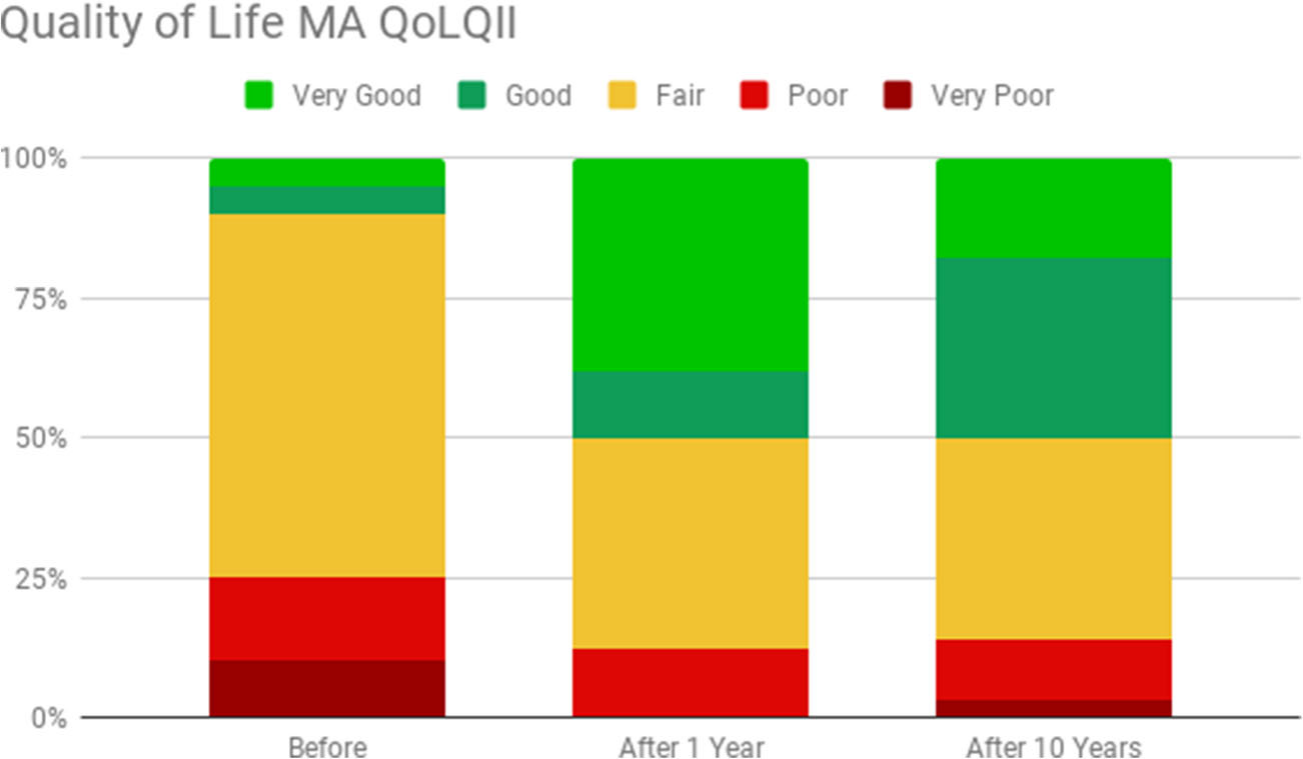
QoL after bariatric surgery according to the MA QoLQII

According to the results of the MA-QoLQII before the surgery, 10% of patients had QoL assessed as “very bad,” 15% as “poor,” 65% as “fair,” 5% as “good,” and 5% as “very good.” One year after the operation, 0% of patients had QoL assessed as “very bad,” 12% as “poor,” 38% as “fair,” 12% as “good,” and 38% as “very good.” At the long-term follow-up 10 years after the bariatric operation, 3% of patients had QoL assessed as “very bad,” 11% as “poor,” 36% as “fair,” 32% as “good,” and 18% as “very good.” Results of the MA-QoLQII are presented in Fig. 4.

## Discussion

Our study focused on long-term outcomes, including weight loss and improvement of QoL, of the two most commonly performed bariatric procedures [2, 20]. QoL was assessed with the SF-36 and MA-QoLQII questionnaires, which are very often incorporated into studies evaluating QoL outcomes after bariatric surgery [21, 22]. This study aimed to verify the long-term effectiveness of bariatric and metabolic surgery in terms of improving the QoL among patients suffering from morbid obesity.

Although the bariatric surgery contribution to the improvement of QoL in the short-term is currently well established, there is still a need for evaluating very long-term outcomes (10 years or more) [21]. There are few studies presenting very long-term outcomes of LSG, and even fewer studies comparing LSG with LRYGB. In our opinion, when qualifying patients for bariatric treatment, surgeons often focus on short-term effects, whereas long-term improvement of the QoL is the most important outcome for the majority of patients. There is a correlation between weight loss or improvement in obesity-related comorbidities and the increase in health-related QoL. A recent review by Kolotkin and Andersen suggests a further need to conduct longer-term studies to evaluate the durability of the short-term outcomes [22]. Additionally, a review by Colquitt et al. suggests that the majority of published evidence presenting beneficial QoL outcomes after bariatric surgery is currently of very low quality [23].

At present, there are multiple published articles comparing weight loss outcomes after LSG and LRYGB, including randomized controlled trials [24]. Peterli et al. and Salminian et al. reported comparable beneficial results in terms of weight loss 5 years after LSG and LRYGB [25, 26]. At the 1-year follow-up, our results demonstrate superior weight loss after LRYGB, which is consistent with previously published data [27].

Literature on very long-term weight loss after bariatric surgery is limited, especially with data concerning LSG. The majority of articles present outcomes of bariatric operations 5 years or more after surgery focusing only on a single procedure [28–30]. According to Kowalewski et al. and Mandeville et al., patients after LSG, in a very long observation (6 to 10 years), achieved a %EWL of 51–60% [28, 31]. In our study, patients achieved a comparable mean %EWL of 45.4% in the very long-term follow-up. Although this outcome is relatively low, there are studies reporting similar results. For instance, Sadot and Spivak reported a %EWL of 53% 20 years after LRYGB [32]. It is important to remember that the patients in our group did not undergo any additional bariatric revisional procedures. In the case of LRYGB, other authors reported %EWL between 58.5 and 78% at 10-year follow-up and %TWL between 26 and 28% [29, 30, 33, 34]. Mantziari et al. reported no significant difference in weight loss between patients < 40 years old, 40–55 years old, and > 50 years old (32.2%, 32.9%, and 32.3% of TWL, respectively) at a 10-year follow-up [35]. Our results seem to be consistent with the abovementioned research, with a mean %EWL of 62.2% and a mean %TWL of 36.5% at a 10-year follow-up examination. There are few studies comparing weight loss outcomes of LSG and LRYGB after more than 5 years. In our study group, patients after LRYGB sustained superior weight loss over a very long-term period compared with patients in the LSG group, which also seems to be consistent with the existing data [36].

Our results present a large improvement of QoL 1 year after bariatric surgery in physical as well as in mental aspects, which seem to be consistent with previously published research. Studies by Amichaud et al. and Charalampakis et al., which presented short-term QoL outcomes of bariatric surgery, reported satisfactory results with significant improvement of QoL after LSG [37, 38]. Poelemeijer et al. reported significant improvement in the majority of QoL domains compared with reference values 1 year after bariatric surgery. Improvement was comparable between LSG and LRYGB, except for physical functioning and general health perception for which RYGB was more beneficial [39]. In the short-term follow-up (1–2 years), authors report comparable outcomes with significant improvement of QoL after both LSG and LRYGB [40, 41]. A study by Takemoto et al. reported significant improvement in both mental and physical aspects of QoL 1 year after bariatric procedures, which remained stable during the following 5 years [42]. Our results did not show a significant difference in QoL improvement between groups undergoing LSG an LRYGB 1 year after surgery.

It was established using observational studies that bariatric procedures after a very long-term may be associated with various problems, including recurrence of morbid obesity or new ailments resulting from complications of the operations. LSG has increased in popularity more recently, and there are very few reports of LSG outcomes with a follow-up of 10 years or longer; available long-term studies investigating QoL focus mostly on LRYGB. Findings included in a meta-analysis by Driscoll et al. present a substantial and significant improvement up to 25 years after the initial bariatric operation [43]. There is scarcely any research on the influence of LSG on QoL 10 years or more after the operation. According to Csendes et al., patients who underwent LSG can develop hiatal hernia, erosive esophagitis, and GERD after a long term (10.5 years), which can potentially influence their QoL [44]. LRYGB, however, has been a popular operation for a long time, and multiple articles present very long-term (10 years or more) outcomes after this procedure. Raoof et al. presented a favorable impact of LRYGB on QoL at a long-term follow-up (7–17 years) in comparison with controls [13]. According to a study by Mantziari et al., the 10-year mean Moorehead–Ardeldt scores were not significantly different between age groups: 1.67 for patients < 40 years old, 1.66 for a group between 40 and 55 years old, and 1.64 for patients > 55 years old [35]. The reported outcomes of LRYGB are mostly favorable, although it seems that this operation is also associated with several long-term complications, for instance, internal or incisional hernia, gallstones, or nutritional deficiencies [45]. Developing such complications would significantly influence QoL; however, this subject requires further investigation. In our study, QoL remained significantly improved at a long-term follow-up; however, there was a slight decrease in QoL between the first and second post-surgery follow-up examinations. Moreover, our data demonstrate that improvement of QoL in patients after LSG may be more sustainable in the long term compared with patients who undergo LRYGB. Unfortunately, in our study group, there was a higher number of patients who were lost to follow-up in LRYGB group compared with the LSG group, which may have affected the statistical significance of the results observed in the LRYGB patients. However, the results of a regression analysis did not reveal potential intergroup differences influencing changes in the QoL.

Surgical treatment of obesity is a physical intervention, and it affects physical QoL to the greatest extent. Patients may require additional psychological care after surgery to regain their mental QoL [21]. Improvement of QoL can have a beneficial impact on multiple aspects of life, including occupational outcomes such as employment or reduction of annual sick days; therefore, improvement of QoL should be regarded as a major goal of bariatric surgery [46]. Knowledge of potential long-term outcomes (including QoL improvement) after each operation should be considered during qualification for bariatric surgery.

Further research investigating the long-term influence of bariatric surgery on QoL should be conducted on a larger study group. It would also be desirable to include analysis of obesity-related comorbidities as factors contributing to QoL.

## Limitations

Our study has several limitations. Unfortunately, the small sample size and the loss of some patients to follow-up (27.7%) are potentially introducing bias and influencing the validity of the results. Although, there was no significant difference in the patients’ baseline characteristics, it may be the result of the small study group. It is also important to note that there were more patients who were lost to follow-up in the LRYGB group than in the LSG group, which may have affected the statistical significance of the results in the LRYGB patients. Therefore, the results of the LSG and LRYGB comparison should be interpreted with caution. In our study group, patients were lost to follow-up due to distant relocation or changing contact data (phone number and address), which prevented us from arranging 10-year follow-up visits. However, considering the very long-time follow-up regimen included in our study design, the loss to follow-up rate was relatively minor. In similar studies, conducted for a long period of time, the follow-up rate is between 26 and 87% [47, 48].

Unfortunately, there were no additional re-evaluations of QoL between years 1 and 10, because our research was not initially planned for such a long period. The QoL may be additionally influenced by aging, which potentially lowers the score 10 years after the surgery.

There was no possibility to include data on improvement or remission of obesity-related comorbidities after bariatric treatment. These data would provide a more comprehensive insight into the long-term outcomes of the evaluated procedures. However, follow-up examinations were conducted exclusively by surgical staff in our center, and assessment of the severity level of each comorbidity would result in deficient data.

## Conclusion

LSG led to significant, durable weight loss and a substantial improvement of QoL in a 10-year follow-up.
